# Endosymbiotic theories for eukaryote origin

**DOI:** 10.1098/rstb.2014.0330

**Published:** 2015-09-26

**Authors:** William F. Martin, Sriram Garg, Verena Zimorski

**Affiliations:** Institute for Molecular Evolution, Universität Düsseldorf, Universitätsstraße 1, Düsseldorf 40225, Germany

**Keywords:** endosymbiosis, eukaryotes, nucleus, mitochondria, plastids, anaerobes

## Abstract

For over 100 years, endosymbiotic theories have figured in thoughts about the differences between prokaryotic and eukaryotic cells. More than 20 different versions of endosymbiotic theory have been presented in the literature to explain the origin of eukaryotes and their mitochondria. Very few of those models account for eukaryotic anaerobes. The role of energy and the energetic constraints that prokaryotic cell organization placed on evolutionary innovation in cell history has recently come to bear on endosymbiotic theory. Only cells that possessed mitochondria had the bioenergetic means to attain eukaryotic cell complexity, which is why there are no true intermediates in the prokaryote-to-eukaryote transition. Current versions of endosymbiotic theory have it that the host was an archaeon (an archaebacterium), not a eukaryote. Hence the evolutionary history and biology of archaea increasingly comes to bear on eukaryotic origins, more than ever before. Here, we have compiled a survey of endosymbiotic theories for the origin of eukaryotes and mitochondria, and for the origin of the eukaryotic nucleus, summarizing the essentials of each and contrasting some of their predictions to the observations. A new aspect of endosymbiosis in eukaryote evolution comes into focus from these considerations: the host for the origin of plastids was a facultative anaerobe.

## 1. Introduction

Early evolution is an important part of life's history, and the origin of eukaryotes is certainly one of early evolution's most important topics, as the collection of papers in this special issue attests. There are various perspectives from which eukaryote origins can be viewed, including palaeontological evidence [1], energetics [2], the origin of eukaryote-specific traits [3,4] or the relationships of the different eukaryotic groups to one another [5]. This paper will look at eukaryote origins from the standpoint of endosymbiotic theory, and how different versions of endosymbiotic theory tend to square off with the data that we have for eukaryotic anaerobes and with regard to data from gene phylogenies. Endosymbiotic theory has a long and eventful history, virtuously summarized in Archibald's book [6], and speaking of history, here is a good place to dispel a myth—about Altmann.

One can occasionally read (though we will politely provide no examples) that Altmann [7] is to be credited with the idea of symbiotic theory for the origin of mitochondria, but that is incorrect. Those of us who can read German and who have a copy of Altmann's 1890 book can attest: in the 1890 book, Altmann was not interested in mitochondria, and he did not propose their symbiotic origin. He mentioned neither mitochondria (nor their older name, chondriosomes) nor endosymbiosis in his book on ‘bioblasts’. To Altmann, everything in eukaryotic cells consisted of bioblasts, including the cytosol, the nucleus and the chromosomes. His bioblasts corresponded to a chemical organization state of matter that was larger than the molecule but smaller than the cell ‘the smallest morphological unit of organized material’ (‘*die kleinste morphologische Einheit der organisirten Materie*’) [8, p. 258]. They would maybe correspond in size roughly to what we today call macromolecular complexes, which however cannot be seen in the light microscopes of Altmann's day. He also distinguished autoblasts, cytoblasts, karyoblasts and somatoblasts, which are mentioned far less often than bioblasts. A scholarly treatise of Altmann in the context of symbiotic theory, and why he cannot be credited with having suggested endosymbiotic theory, can be found in Höxtermann & Mollenhauer [8].

The concept of symbiosis (Latin, ‘living together’), that two different organisms can stably coexist and even give rise to a new type of organism, traces to Simon Schwendener [9], a Swiss botanist who discovered that lichens consist of a fungus and a photosynthesizer. The German botanist Heinrich Anton de Bary (1878) coined the term ‘*Symbiose*’ to designate this type of coexistence [10]. Schimper [11] is sometimes credited with the discovery of endosymbiotic theory, but his treatise of the topic is wholly contained in a footnote that translates to this: ‘If it can be conclusively confirmed that plastids do not arise de novo in egg cells, the relationship between plastids and the organisms within which they are contained would be somewhat reminiscent of a symbiosis. Green plants may in fact owe their origin to the unification of a colorless organism with one uniformly tinged with chlorophyll’ [11, pp. 112–113]. That was all he wrote on the possibility of symbiotic plastid origin. The sentence immediately following that one in Schimper's famous footnote, however, is also significant, as we will see in a later passage about Portier and the symbiotic origin of mitochondria; it translates to this: ‘According to Reinke (Allg. Botanik, p. 62) the chlorophyll bodies [Chlorophyllkörner, another name for plastids in Schimper's day] might even have the ability to live independently; he observed this phenomenon, as communicated to me, and published with kind permission, in a rotting pumpkin, the chloroplastids of which, surrounded by Pleosporahyphae, continued to vegetate in dead cells and multiplied by division’ [11, p. 113]. Clearly, Reinke was observing the proliferation of contaminating bacteria, not of free-living organelles.

Schimper [11,12] did, however, champion the case that plastids proliferate through division. That was important for the Russian biologist Constantin Mereschkowsky, who probably delivered the first thoroughly argued case that some cells arose through the intracellular union of two different kinds of cells (endosymbiosis), in his 1905 paper [13] that has been translated into English [14]. Mereschkowsky [13] said three things: (i) plastids are unquestionably reduced cyanobacteria that early in evolution entered into a symbiosis with a heterotrophic host, (ii) the host that acquired plastids was itself the product of an earlier symbiosis between a larger, heterotrophic, amoeboid host cell and a smaller ‘micrococcal’ endosymbiont that gave rise to the nucleus, and (iii) the autotrophy of plants is an inheritance, *in toto*, from cyanobacteria [13].

Mereschkowsky's scheme was more fully elaborated but basically unchanged in his 1910 series [15]: there were two kinds of fungi, those that evolved a nucleus without endosymbiosis and those that once possessed plastids but became secondarily non-photosynthetic, today we call them the oomycetes, and there is still no consensus on the issue of whether they ever had plastids or not. The branches in Mereschkowsky's tree occasionally unite via endosymbiosis to produce fundamentally and radically new kinds of organisms (plants, for example) [15,16]. A more modern version of symbiosis in cell evolution would have to include the symbiotic origin of mitochondria, archaea and the concept of secondary endosymbiosis. Endosymbiotic theories have it that cells unite, one inside the other, during evolution to give rise to novel lineages at the highest taxonomic levels, via combination. That is not the kind of evolution that Darwin had in mind; his view of evolution was one of gradualism.

Many biologists still have a problem with the notion of endosymbiosis and hence prefer to envisage the origin of eukaryotes as the product of gene duplication, point mutation and micromutational processes [17]. A 2007 paper by the late Christian de Duve [18] is now often taken as the flagpole for micromutational theories of eukaryote origin, but de Duve, like the late Lynn Margulis [19], always categorically rejected the evidence that mitochondria and hydrogenosomes—anaerobic forms of mitochondria [20,21]—share a common ancestor. No anaerobic form of mitochondria ever fits into classical endosymbiotic theory. This is because classical (Margulis's version of) endosymbiotic theory [19] was based on the premise that the benefit of the endosymbiotic origins of mitochondria was founded in oxygen utilization, while de Duve's versions went one step further and suggested that even the endosymbiotic origin of peroxisomes was founded in oxygen utilization [18]. Anaerobic mitochondria were never mentioned and hydrogenosomes, if they were mentioned, were explained away as not being mitochondria [18,19]. The overemphasis of oxygen in endosymbiotic theory and how the focus on oxygen led to much confusion concerning the phylogenetic distribution and evolutionary significance of anaerobic forms of mitochondria has been dealt with elsewhere [22–24].

There is one alternative to classical endosymbiotic theory that took anaerobic mitochondria and hydrogenosomes into account, the hydrogen hypothesis [25]; it predicted (i) all eukaryotes to possess mitochondria or to have secondarily lost them, (ii) that the host for mitochondrial origins was an archaeon, the eukaryotic state having arisen in the wake of mitochondrial origins, and (iii) that aerobic and anaerobic forms should interleave on the eukaryotic tree. Though radical at the time, prediction (i) was borne out [26–29], and so was prediction (ii) [30–32], as well as (iii) [21,33]. Furthermore, only recently, it has been recognized that the invention of eukaryotic specific traits required more metabolic energy per gene than prokaryotes have at their disposal, and that mitochondria afforded eukaryotic cells an orders of magnitude increase in the amount of energy per gene, which (finally) explains *why* the origin of eukaryotes corresponds to the origin of mitochondria [2,34]. But there is more to eukaryote origins than just three predictions and energy. There is the origin of the nucleus to deal with [35], and the role that gene phylogenies have come to play in the issues. In addition, there is the full suite of characters that distinguish eukaryotes from prokaryotes to consider (meiosis, mitosis, cell cycle, membrane traffic, endoplasmic reticulum (ER), Golgi, flagella and all the other eukaryote-specific attributes, including a full-blown cytoskeleton—not just a spattering of prokaryotic homologues for cytoskeletal proteins [31]), but here our focus is on endosymbiotic theories, not the autogenous origin of ancestrally shared eukaryotic characters, whose origins for energetic reasons come in the wake of mitochondrial origin [34].

## 2. Gene trees, not as simple as it sounds

To get a fuller picture of eukaryote origins, we have to incorporate lateral gene transfer (LGT) among prokaryotes, endosymbiosis and gene transfer from organelles to the nucleus into the picture. That is not as simple as it might seem, because it has become apparent that individual genes have individual and differing histories. Thus, in order to get the big picture, we would have to integrate all individual gene trees into one summary diagram in such a way as to take the evolutionary affinities of the plastid (a cyanobacterium), the mitochondrion (a proteobacterium) and the host (an archaeon) into account. Nobody has done that yet, although there are some attempts in that direction [36]. In 2015, our typical picture of eukaryotic origins entails either a phylogenetic tree based on one gene or, more commonly now, a concatenated analysis of a small sample of genes (say 30 or so from each genome), which generates a tree, the hope being that the tree so obtained will be representative for the genome as a whole and thus will have some predictive character for what we might observe in phylogenies beyond the 30 or so genes used to make the tree. The 30 or so genes commonly used for such concatenated phylogenies are mostly ribosomal proteins or other proteins involved in information processing, genes that Jim Lake called informational genes in 1998 [37].

But because of the role of endosymbiosis in eukaryote cell evolution, eukaryotes tend to have two evolutionarily distinct sets of ribosomes (archaeal ribosomes in the cytosol and bacterial ribosomes in the mitochondrion), or sometimes three (an additional bacterial set in the plastid [38]) and in rare cases four sets of active ribosomes (yet one more set in algae that possess nucleomorphs) [39]. The ‘core set of genes’ approach, in all of its manifestations so far, only queried cytosolic ribosomes for eukaryotes, and thus only looked at the archaeal component of eukaryotic cell history. Some of us have been worried that by looking only at genes that reflect the archaeal component of eukaryotic cells we might be missing a lot, because it was apparent early on that many genes in eukaryotes do not stem from archaea, but from bacteria instead and, most reasonably under endosymbiotic theory, from organelles [40,41].

An early study looking at the phylogeny of the core gene set, which largely but not entirely corresponds to the ribosomal protein superoperon of prokaryotes, came to the conclusion that the information contained within the alignment is problematic because of the low amount of sequence conservation involved across many of the sites [42]. Concerns were also voiced that the 30 genes of the set, if analysed individually, might not have the same history and that concatenation might thus be a problem [43], but that did not stop bioinformaticians [44] from rediscovering the same set of 30 or so genes and making a tree that looked remarkably similar to the rRNA tree in most salient aspects, in particular as regards the position of the eukaryotes. By that time it was reasonably well-known that the genes of archaeal origin in eukaryotes are not representative of the genomes as a whole; they constitute a minority of the genome and are vastly outnumbered by genes of bacterial origin [45]. Despite that, the attention in the issue of eukaryote origins has, with few exceptions [46–48], remained focused on the archaeal component, and it will probably stay that way until improved methods to summarize the information contained in thousands of trees come to the fore.

Always critical of the branches in trees that phylogenetic methods produce [49], Embley and colleagues looked at the conserved core set with more discerning phylogenetic methods [30,50,51] and found that the archaeal component of eukaryotes branches within the archaea. These new trees tend to group the eukaryotes with the crenarchaeotes, specifically with the TACK superphylum of archaea [31], while at the same time tending to locate the root of the archaea among the euryarchaeotes, sometimes among the methanogens [52].

Now is a good time to have a look at endosymbiotic theories and related ideas for the origin of eukaryotes, their nucleus and their mitochondria. In doing so, we pick up on our own earlier reviews of the topic [22,53], the figures of which have become popular [31]. In the next section, we summarize what various models say, starting with models for the origin of the nucleus, and then move on to models for the origins of chloroplasts and mitochondria.

## 3. The nucleus

**Figure 1.**
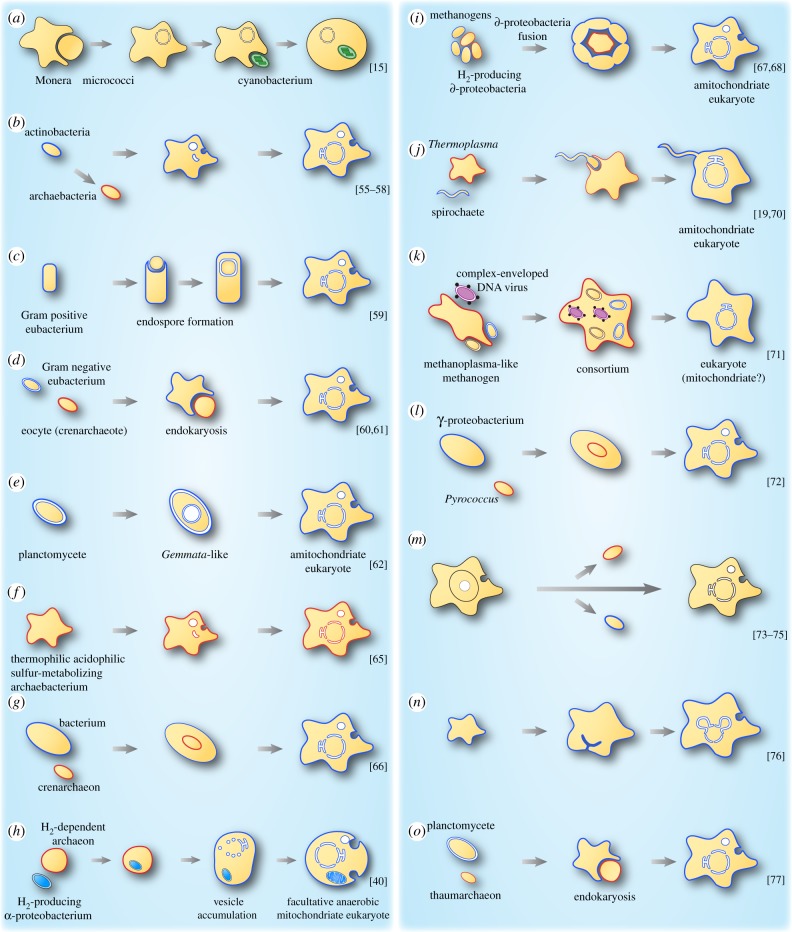
Models describing the origin of the nucleus in eukaryotes. (*a*–*o*) Schematic of various models accounting for the origin of the nucleus. Archaeal cells/membranes are represented with red, while blue indicates eubacterial cells/membranes. Black membranes are used when the phylogenetic identity of the cell is not clear or not specified. See also [22,53].

The nucleus is a defining feature of eukaryotes [54]. Theories for the evolution of the nucleus are usually based (i) on invaginations of the plasma membrane in a prokaryote or (ii) on endosymbiosis of an archaeon in a eubacterial host or (iii) on an autogenous origin of a new membrane system including the nuclear envelope in a host of archaeal origin after acquisition of mitochondria. The endosymbiotic theory for the origin of the nucleus started with Mereschkowsky [13]. He postulated that the nucleus evolved from a prokaryote (mycoplasma), which was engulfed by an amoeboid cell homologous to the eukaryotic cytosol (figure 1*a*; [15]).

Cavalier-Smith argued that nuclear and ER membranes originated through invaginations of the plasma membrane of a prokaryotic cell (figure 1*b*; [55–58]). He suggested that the prokaryote initially lost its cell wall and thereby gained the ability to phagocytose food particles. Ribosomes, primarily attached to the plasma membrane, became internalized, but still attached to the membrane, resulting first in the rough ER and out of it the nuclear envelope. Gould & Dring [59] presented a different model in 1979 where they described that endospore formation of Gram-positive bacteria resulted in the origin of the nucleus. The protoplast of a single cell divides during endospore formation in such a manner that the cell engulfs a portion of its own cytoplasm, which than becomes surrounded by a double membrane resulting in the cell's nucleus (figure 1*c*; [59]). In the 1990s, several models for the origin of the nucleus via endosymbiosis (sometimes called endokaryotic theories) were published, but only few refer to Mereschkowsky's original suggestion. They have in common that they envisage a eubacterial host that engulfed an archaebacterial endosymbiont that underwent a transformation into the nucleus (figure 1*d*; [60,61]). Fuerst & Webb [62] observed that the DNA in the freshwater budding eubacterium *Gemmata obscuriglobus* (a member of the *Planctomyces-Pirella* group) appears to be surrounded by a folded membrane, the organization of which was thought to resemble the nucleus (figure 1*e*; [62]). Later papers were less cautious and called this structure a nucleus outright [63]; subsequent work on *Gemmata* showed that the inner membrane is simply an invagination of the plasma membrane [64], as had been previously pointed out [53]. Searcy & Hixon [65] interpreted thermophilic acidophilic sulfur-metabolizing archaebacteria lacking a rigid cell wall but having a well-developed cytoskeleton as a primary stage for the evolution of eukaryotic cells (figure 1*f*; [65]).

Lake & Rivera [66] suggested an endosymbiosis in which a bacterium engulfed an archaeon (crenarchaeon) for the origin of eukaryotes (figure 1*g*). A vesicular model for the origin of the nucleus in a cell that had a mitochondrial endosymbiont was proposed (figure 1*h*; [40]). It posits a role for gene transfer and the origin of bacterial lipids in the origin of the eukaryotic endomembrane system, and in a subsequent formulation [35] it posits a causal relationship between the origin of spliceosomes and the origin of nucleus–cytosol compartmentation (this aspect is discussed in more detail in a later section). Moreira & López-García [67,68] modified the endokaryotic model, invoking the principle of anaerobic syntrophy (H_2_-dependence) for the origin of the nucleus. They postulated a fusion of plasma membranes in an agglomeration of δ-proteobacteria entrapping a methanogenic archaebacterium, which evolved to the nucleus (figure 1*i*; [67,68]). The kind of fusion of plasma membranes among free-living cells that Moreira & Lopez-Garcia [67,68] envisage has not been observed for bacteria, but it is known to occur among archaea [69]. Lynn Margulis presented another symbiogenic theory for the origin of the nucleus. She suggested a symbiosis between a spirochaete and an archaebacterium without a cell wall (most likely *Thermoplasma*-like in her view), leading to both the eukaryotic flagellum and the nucleus (figure 1*j*; [19,70]). A viral origin for the nucleus involving poxviruses was suggested in 2001 by Bell in the context of syntrophic consortia involving methanogens (figure 1*k*; [71]). Horiike postulated a model in which the nucleus emerged from an archaeal endosymbiont (*Pyrococcus*-like), which was engulfed by a *γ*-proteobacterium (figure 1*l*; [72]). An origin of eukaryotes (hence implicitly or explicitly their nucleus) prior to prokaryotes has also been repeatedly suggested (figure l*m*; [73–75]). Penny argues that prokaryotes, which he and Forterre [73] sometimes call ‘akaryotes’ [75], arose from this eukaryote ancestor via Forterre's thermoreduction hypothesis—a transition to the prokaryotic state from a eukaryotic ancestor in response to higher temperatures.

More recently, the community of scientists interested in cytoskeletal evolution have—in unaltered form—rekindled Cavalier-Smith's hypothesis of an autogenous (non-symbiotic) origin of a phagocytosing amitochondriate eukaryote (an archezoon) via point mutational changes leading to a host that does not need a mitochondrion at all to enjoy its phagocytotic lifestyle, but acquires one nonetheless (figure 1*n*; [76]).

Forterre [77] departed from thermoreduction and introduced a new variant of the endokaryotic hypothesis, one that got planctomycetes (a member of the PVC group: Planctomycetes, Verrucomicrobia, Chlamydiae) involved in eukaryote origin as the bacterial host for the engulfment of a thaumarchaeon as the nucleus, followed by invasions of retroviruses and nucleo-cytoplasmic large DNA viruses (NCLDV). In this theory, the PTV (for PVC–thaumarchaeon–virus) fusion hypothesis, the PVC bacterium provides universal components of eukaryotic membranes required also for the formation of the nucleus and the thaumarchaeon provides informational and operational proteins and precursors of the modern eukaryotic cytoskeleton and vesicle trafficking system (figure 1*o*; [77]).

A problem with all models that envisage a role for planctomycetes in eukaryote origin is that there is no molecular phylogenetic evidence that would link any lineage of planctomycetes with eukaryotes [78]. The problems with theories that derive the nucleus from an endosymbiont are numerous and have been listed in detail elsewhere [40]; in essence, they fail to explain why the nuclear compartment is so fundamentally different from any free-living cell from the standpoints of (i) biosynthetic or ATP-generating physiology (altogether lacking in the nuclear compartment), (ii) membrane topology (no free-living cell is bounded similarly), (iii) permeability (no prokaryotic cytosol is contiguous with the environment via pores), and (iv) division (dissolution of a superficial homologue to the plasma membrane once per cell division in eukaryotes with open mitosis). Endosymbiotic theories for plastid and mitochondrial origin do not have those problems. A problem with the thermoreduction hypothesis is that it does not address the issue of where eukaryotes come from in the first place, it just takes their origin as a given. The recognition that the common ancestor of eukaryotes possessed a mitochondrion [30,32,79] is a severe problem for thermoreduction hypotheses, because the eukaryote has to first give rise to a prokaryote (the mitochondrial ancestor) that is required for its own origin, a sequence of events that, at face value, requires time to run backwards. Thermoreduction hypotheses are generally silent regarding the origin of mitochondria. Very few models for the origin of the nucleus, possibly only one, derive the nucleus in an archaeal host that possessed a mitochondrion. That model posits the nuclear membrane to arise from vesicles of membranes consisting of bacterial lipids [40] and invokes the need to separate splicing from translation as the selective pressure that led to the fixation of the compartmentation into nucleoplasm and cytoplasm [35].

The recent focus both on the evolution of cytoskeletal components [76] and on an autogenous (non-symbiotic) origin of a phagocytosing amitochondriate eukaryote point to a problem that should be mentioned. That theory, once called the archezoa hypothesis [55,56], now sometimes called the phagocytosing archaeon theory [31], envisages that point gradual changes lead to a prokaryotic host that can perform fully fledged eukaryotic phagocytosis (a quite complex process). These theories have it that phagocytosis is the key character that enabled the endosymbiotic origin of mitochondria. A problem common to those theories is that the phagocytotic, primitively amitochondriate eukaryote does not need a mitochondrion at all, and if there were some construable selective advantage then eukaryotes should have arisen from prokaryotes in multiple lineages independently. That has always been one of the weakest aspects of autogenous theories, in addition to the bioenergetic aspects [34].

## 4. The origin of mitochondria (and chloroplasts)

**Figure 2.**
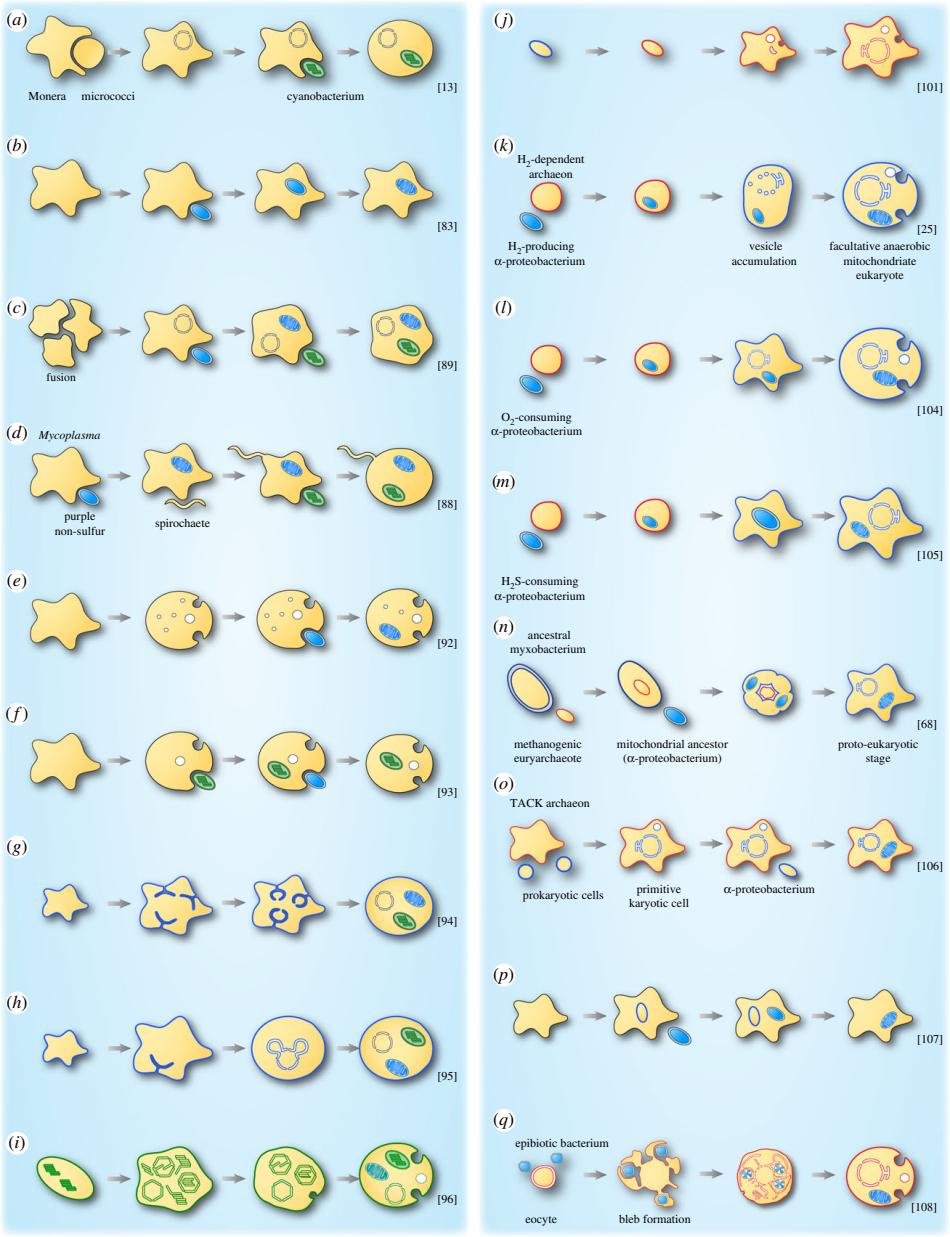
Models describing the origin of mitochondria and/or chloroplasts in eukaryotes. (*a*–*q*) Schematic of various models accounting for the origin of mitochondria and/or chloroplasts. Archaeal cells/membranes are represented with red, while blue indicates eubacterial cells/membranes. Black membranes are used when the identity of the cell is not clear and green is used for cyanobacterial derived cells/membranes. See also [22].

Endosymbiotic theory for the origin of chloroplasts and mitochondria started again with Mereschkowsky [13] and his idea about a symbiosis between ‘chromatophores’ (plastids) and a heterotrophic amoeboid cell. He contradicted the orthodox view that chromatophores are autogenous organs of the plant cells; he saw them as symbionts, extrinsic bodies or organisms, which entered into the host's plasma establishing a symbiotic relationship. The host for the origin of plastids itself originated, in his view, from an earlier symbiosis between a heterotrophic, amoeboid cell and a ‘micrococcal’ endosymbiont that gave rise to the nucleus (figure 2*a*; [13]). Comparison of physiological and anatomic attributes of plastids and cyanobacteria known at that time led him to the certain conclusion that the endosymbionts were ‘cyanophyceae’ (cyanobacteria) that entered into symbioses with amoeboid or flagellated cells on several independent occasions, leading to a plant kingdom having several independent origins. That is, he viewed the different coloured plastids of algae (red, green, brown, golden) as inheritances from different endosymbionts, each having those different pigmentations. Although he was wrong on that specific interpretation—today there is broad agreement that the plastids of all plants and algae have a single origin [80–82]—he was right with the endosymbiotic, cyanobacterial origin of plastids.

Mereschkowsky failed, however, to recognize the endosymbiotic origin of mitochondria, although the physiological properties of cells that he explained with the endosymbiotic origin of the nucleus are, from today's perspective, properties of mitochondria [15]. As very readably explained by Archibald [6], Portier developed (in French) the idea that there was a close relationship between bacteria and mitochondria and that mitochondria were involved in numerous processes in the cell. But like Schimper in his footnote regarding plastids, which we translated above, Portier proposed that mitochondria could be cultured outside their host cells, and this precipitated unforgiving criticism from his contemporaries [6]. Clearly, both Reinke (as cited in Schimper's footnote that we translated above) and Portier were observing the proliferation of contaminating bacteria, not of free-living organelles. Wallin [83] developed the endosymbiotic theory further for mitochondria, in English. He recognized that these organelles are descendants of endosymbiotic bacteria, but it remained very unclear what his idea about the host was (figure 2*b*; [83]). Like Portier, he thought the cultivation of mitochondria outside their host to be possible. But he had the concept of gene transfer from organelles to the nucleus in mind: ‘It appears logical, however, that under certain circumstances, […] bacterial organisms may develop an absolute symbiosis with a higher organism and in some way or another impress a new character on the factors of heredity. The simplest and most readily conceivable mechanism by which the alteration takes place would be the addition of new genes to the chromosomes from the bacterial symbiont’ [84, p. 144].

In print, cell biologists rejected endosymbiotic theory during the 1920s and through into the 1970s. A few prominent trouncings were (i) from Wilson [85] who wrote (pp. 738–739) ‘Mereschkowsky (‘10), in an entertaining fantasy, has developed the hypothesis’ … ‘in further flights of the imagination Mereschkowsky suggests’, (ii) from Buchner [86] (pp. 79–80), who discussed endosymbiotic theory in a chapter entitled ‘*Irrwege der Symbioseforschung*’ (translation: Symbiosis research gone astray) and (iii) from Lederberg [87], who surmised (p. 424): ‘We should not be too explicit in mistaking possibilities for certainties. Perhaps the disrepute attached to some of the ideas represented in this review follows from uncritical over-statements of them, such as the Famintzin–Merechowsky theory of the phylogeny of chloroplasts from cyanophytes (28, 126) or the identity of mitochondria with free-living bacteria (198)’.

Endosymbiotic theory was repopularized in 1967 by Lynn Sagan (later Margulis) [88] and also mentioned in a very curious paper by Goksøyr [89]. As far as we can tell, those were the initial suggestions in endosymbiotic theory that both chloroplasts and mitochondria are descended from endosymbionts, but from separate endosymbionts. Goksøyr suggested an evolutionary development of mitochondria and later, in an independent symbiosis, chloroplasts from prokaryotic forms through a coenocytic relationship in which anaerobic prokaryotes (most likely of a single species) were brought into contact without intervening cell walls (figure 2*c*; [89]). The DNA of these cells accumulated in the centre of the agglomerate, a nuclear membrane arose from an endoplasmic reticulum, establishing an anaerobic eukaryotic cell. Aerobic eukaryotes trace back to an endocellular symbiotic relationship of anaerobic eukaryotes with aerobic prokaryotes, which emerged with the enrichment of oxygen in the atmosphere. The later loss of autonomy by the aerobic prokaryote to become a mitochondrion came along with gene transfer to the host's nucleus. An uptake of a primitive cyanobacterium, involving gene transfers to the nucleus again, led to photosynthetic eukaryotes. Goksøyr assumed that coenocytic systems occurred several times from different prokaryotic forms, making the origin of eukaryotes a non-monophyletic one [89]. Goksøyr's paper contains only one reference, to a 1964 paper by Stanier, and no mention of the older symbiotic literature.

Lynn Sagan rekindled the idea of a prokaryotic ancestry of mitochondria and chloroplasts and extended the idea to include a spirochaete origin of flagella [88]. On the second page of her 1967 paper, which was reported to have been rejected by 15 different journals [90], she states ‘Although these ideas are not new…’ while referring to Mereschkowsky's 1910 paper [15], although Mereschkowsky does not appear in the bibliography of her 1970 book [91]. She suggested the origin of eukaryotes from prokaryotes to be related to the increasing production of free oxygen by photosynthetic prokaryotes and the increasing proportion of oxygen in the atmosphere. Her host was a heterotrophic anaerobic prokaryote (perhaps similar to *Mycoplasma*), in whose cytoplasm an aerobic prokaryotic microbe (the proto-mitochondrion) was ingested, resulting in the evolution of an aerobic amoeboid organism, which later acquired a spirochaete, resulting in the eukaryotic flagellum (figure 2*d*; [88]; her later versions modified that order of events). She depicted the evolution of plastids as several ingestions of different photosynthetic prokaryotes (protoplastids—evolved from oxygen-consuming prokaryotes, homologous to cyanobacteria) by heterotrophic protozoans (figure 2*d*; [88]).

Countering Margulis, de Duve [92] outlined that the primitive phagocyte, which symbiotically adopted different types of microorganisms, was a primitive aerobe that remained dependent on hydrogen peroxide-mediated respiration during its early evolution, establishing through the loss of the cell wall and the evolution of membrane invagination processes (endocytosis) a primitive phagocyte with peroxisomes as the main (aerobic) respiratory organelle. This amitochondriate, peroxisome-bearing organism became later the host of an aerobic bacterium with oxidative phosphorylation, the ancestor of mitochondria (figure 2*e*; [92]). Stanier suggested an anaerobic, heterotrophic host in the evolution of chloroplasts [93] and placed the origin of chloroplasts before the origin of mitochondria, arguing that since mitochondria use oxygen, and since eukaryote origin took place in anaerobic times, there must have been first a sufficient and continuous source of oxygen before mitochondria were able to develop (figure 2*f*; [93]).

In the early 1970s, there was considerable resistance to the concept of symbiosis in cell evolution. Raff & Mahler [94] presented an alternative, non-symbiotic model for the origin of mitochondria, proposing that the proto-eukaryote was an advanced, heterotrophic, aerobic cell of large size, which enlarged the respiratory membrane surface achieved by invaginations of the inner cell membrane, which then formed membrane-bound vesicles blebbing off the respiratory membrane, generating closed respiratory organelles acquiring an outer membrane later on (compartmentalization, figure 2*g*; [94]). Bogorad [95] described a cluster clone hypothesis for the origin of eukaryotic cells from an uncompartmentalized single cell. He suggested that the cell's genome split into gene clusters (representing a new genome), followed by a membrane development around each gene cluster to create one or more gene-containing structures from which nuclei, mitochondria and chloroplasts evolved (figure 2*h*; [95]). Cavalier-Smith [96] explained the origin of chloroplasts and mitochondria by fusion and restructuring of thylakoids in a cyanobacterium. Plastids resulted through restructuring of photosynthetic thylakoids and mitochondria through restructuring of respiratory thylakoids, respectively (figure 2*i*; [96]). Though molecular evolutionary studies put non-symbiotic models for the origin of plastids and mitochondria more or less out of business [97], skepticism regarding endosymbiotic theory tends to run deep. Anderson *et al.* [98] in their publication on human mitochondrial DNA concluded that the data ‘make it difficult to draw conclusions about mitochondrial evolution. Some form of endosymbiosis, involving the colonization of a primitive eukaryotic cell by a respiring bacteria-like organism, is an attractive hypothesis to explain the origin of mitochondria. However, the endosymbiont may have been no more closely related to current prokaryotes than to eukaryotes’ [98, p. 464].

During the 1970s and 1980s, some other models for the origin of eukaryotes were developed, which are not presented in figure 2. John & Whatley [99] presented a very explicit symbiotic model for the origin of mitochondria with an anaerobic, fermenting, mitochondrion-lacking ‘proto-eukaryote’ as the host for a free-living aerobic respiring bacterium (similar to *Paracoccus denitrificans*), giving rise to the mitochondria where again the host's origin is not addressed. Woese [100] recognized that the archaebacteria might be related to the host lineage in endosymbiotic theory, but his model for the origin of mitochondria suggested a mitochondrial origin early in Earth's history, when the atmosphere was anaerobic, that mitochondria might descend from an initially photosynthetic organelle, that gained the ability of oxygenic respiration after becoming an endosymbiont [100].

In 1980, both van Valen & Maiorana (figure 2*j*; [101]) and Doolittle [102] put archaebacteria into the context of endosymbiosis, suggesting that they are the sister groups of the host that acquired the mitochondrion. Margulis [103] adjusted her version of endosymbiotic theory to accommodate the discoveries of archaea accordingly, but she kept the symbiotic (spirochaete) origin of flagella.

The hydrogen hypothesis posits anaerobic syntrophy as the ecological context linking the symbiotic association of an anaerobic, strictly hydrogen-dependent and autotrophic archaebacterium as the host with a facultatively anaerobic, heterotrophic eubacterium as endosymbiont (figure 2*k*; [25]). It entails an ancestral mitochondrion that could use either its electron transport chain or use mixed acid (H_2_-producing) fermentations, thus it directly accounts for the common ancestry of mitochondria and hydrogenosomes as well as for intermediate forms between the two, the anaerobic mitochondria [21]. The model of Vellai and Vida [104] operates with a prokaryotic host for the origin of mitochondria (figure 2*l*), as does the sulfur cycling theory of Searcy (figure 2*m*; [105]), but neither accounts for hydrogenosomes or anaerobic mitochondria.

López-García & Moreira [68] proposed an evolutionary scenario for the origin of mitochondria that also includes an endosymbiotic origin of the nucleus. Their model is also a syntrophic symbiosis mediated by interspecies hydrogen transfer between a strict anaerobic, methanogenic archaeon, that became the nucleus, and a fermenting, heterotrophic, hydrogen-producing ancestral myxobacterium (δ-proteobacterium) [68] that served as its host; the mitochondrial ancestor (an *α*-proteobacterium) was then surrounded by the syntrophic couple, which led to an obligatory (endo)symbiotic stage with metabolic compartmentation as selective force to avoid interference of opposite anabolic and catabolic pathways. After the mitochondrion was stabilized, a loss of methanogenesis occurred generating the proto-eukaryote stage, in which the archaeal endosymbiont became the nucleus (figure 2*n*; [68]).

The phagocytosing archaeon theory was proposed by Martijn & Ettema [106], which posits an archaeon (most probably belonging to the TACK superphylum) and an α-proteobacterium (the proto-mitochondrion). The archaeon first phagocytotically took up various forms of other prokaryotic cells and digested them, resulting in gene transfers, whereby we note that phagocytosis is not required for gene transfer among prokaryotes. To protect its genetic material from such ‘contamination’ a membrane was formed by invagination (the nuclear envelope), resulting in a primitive karyotic cell type. At that stage, an α-proteobacterium was engulfed, establishing an endosymbiotic interaction with the host, leading to a protomitochondrial cell type (figure 2*o*; [106]). This model that has quite a bit in common with that of Cavalier-Smith [57] in that the origin of eukaryotic cell complexity (phagocytosis and nucleus) preceeds the origin of mitochondria, which for energetic reasons is unlikely [34]. Gray [107] recently proposed the pre-mitochondrion hypothesis, which does not account for the origin of eukaryotes but assumes that the host was already more or less eukaryotic in organization, and furthermore assumes that the host was aerobic prior to the origin of mitochondria, emphasizing, like de Duve & Margulis [18,19], oxygen in endosymbiotic theory. The origin of mitochondria was preceded by an ATP-consuming ‘compartment’, the pre-mitochondrion, presumably surrounded by one membrane (he is not explicit on this point), that became converted into the mitochondrion via retargeting of its proteins into a *Rickettsia*-like α-proteobacterial endosymbiont (figure 2*p*; [107]). The pre-mitochondrion hypothesis is silent on the origin of the archaeal components of eukaryotes, on the presence or the absence of a nucleus in the host, and on anaerobic forms of mitochondria.

The perhaps latest model for the origin of the eukaryotic cell and mitochondria is the inside-out theory by David & Buzz Baum [108]. They argued that an increasing intimate mutualistic association between an archaeal host (eocyte) and an epibiotic α-proteobacterium (the proto-mitochondrion), which initially lived on the host cell surface, drove the origin of eukaryotes. The host cell started to form protrusions and bleb enlargements to achieve a greater area of contact between the symbiotic partners, resulting in the outer nuclear membrane, plasma membrane and cytoplasm, whereas the spaces between the blebs generated the ER. The symbionts were initially trapped in the ER, but penetrated the ER's membrane to localize to the cytosol during evolution (figure 2*q*; [108]).

This section has shown that much thought has been invested on the topic of how the mitochondrial endosymbiont could have entered its host. Many theories place a premium on phagocytosis and predation upon bacteria as the essential step for allowing the symbiont to enter its host. Predation is actually very widespread among bacteria [109], but it never involves phagocytosis, instead it involves *Bdellovibrio*-like penetration mechanisms, an ability that has evolved in many independent lineages of bacteria, including *Micavibrio*, and that has been suggested to have possibly played a role in mitochondrial origin [110,111]. But predation, whether involving phagocytosis or bacterial predation, leaves mitochondria looking like leftovers of indigestion. Endosymbiosis and organelle origins are not about digestion. Microbial symbiosis, the process that gave rise to bioenergetic organelles, is about chemistry.

## 5. Anaerobes and mitochondrial origin in a prokaryotic host

Endosymbiotic theory is traditionally founded in comparative physiology (core carbon and energy metabolism). That is true for Mereschkowsky [13,15], for Margulis's 1970 formulation [91], for John and Whatley's version [99], and for van Valen and Maiorana's version [101]. The only formulation of endosymbiotic theory that directly accounts for anaerobic mitochondria and the (largely phylogeny-independent) distribution of anaerobes across all major eukaryotic groups and their use of the same small set of enzymes underlying their anaerobic ATP synthetic pathways [21] is the hydrogen hypothesis, which is also founded in comparative physiology.

The theories in the foregoing have different strengths and weaknesses; they are also designed to explain different aspects of eukaryotic cells too numerous to outline here. It is not our aim to defend them all or criticize them all. Instead we wish to focus on one of them, the one that accounts for the anaerobes. Theories are supposed to make testable predictions; in that respect the hydrogen hypothesis [25] has done fairly well. It posits that the host for the origin of mitochondria (hereafter, the host) was an archaeon, not a eukaryote, a view that is now current [30,31]. It predicted that no eukaryotes are primitively amitochondriate. That view is now conventional wisdom on the issue [28,30,32,33], though it was far from common wisdom when proposed. Other theories ultimately generated the same prediction with regard to mitochondrial ubiquity but were not explicit on organisms like *Entamoeba*, *Giardia* and microsporidia, which harbour neither respiring mitochondria nor fermenting hydrogenosomes and were later found to harbour relict organelles that came to be known as mitosomes [26,27,112–114]. The hydrogen hypothesis did not directly predict the existence of mitosomes, but it did explicitly predict that organisms like *Entamoeba* and *Giardia* are derived, via reduction, from organisms that possessed the same endosymbiont as gave rise to mitochondria and hydrogenosomes. It also clearly predicted the chimaeric nature of eukaryotic genomes [32], which well into the late 1990s were supposed to represent a pure archaeal lineage [115].

**Figure 3.**
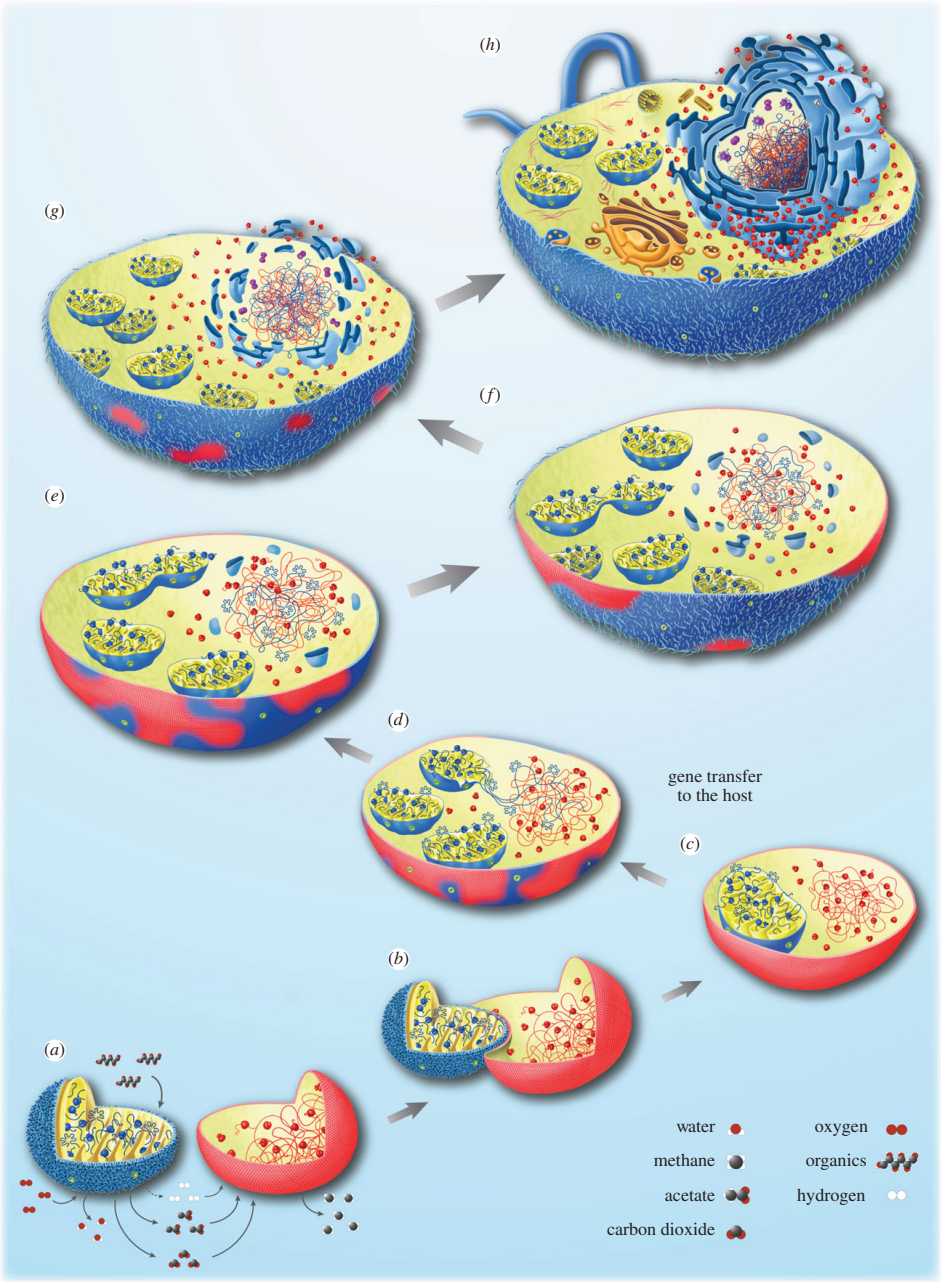
Mitochondrial origin in a prokaryotic host. (*a*–*h*) Illustrations for various stages depicting the transition of a H_2_-dependent archaeal host (in red) and a facultatively anaerobic *α*-proteobacterium (in blue) to an eukaryote. See also [25,34,35] regarding this transition, and [116,117] regarding gene transfer from organelles to the nucleus.

The nature of host–symbiont interactions at the onset of mitochondrial symbiosis in the hydrogen hypothesis was posited to be anaerobic syntrophy, the host being a H_2_-dependent archaeon, the symbiont being a facultative anaerobe that was able to respire in the presence of O_2_, or to perform H_2_-producing fermentations under anaerobic conditions. This is sketched in figure 3*a* for the example of methanogenesis, the metabolic model upon which the hypothesis was based, but, clearly, there are many H_2_-dependent archaea, and it was clearly stated that any strictly H_2_-dependent host would fit the bill [25]. This is the strength of the hydrogen hypothesis, because its host actually needs its mitochondrial symbiont. This is not true for any other version of endosymbiotic theory. Variants have been proposed that invoke anaerobic syntrophy to derive the nucleus via endosymbiosis [67,68,118], but they posit no metabolic demand or requirement for the involvement of mitochondria at eukaryote origin. In all versions of the endosymbiont hypothesis that entail a heterotrophic host, the host does not need its (mitochondrial) endosymbiont.

Anaerobic syntrophy (H_2_-transfer) is thus the metabolic context of host–symbiont association, leading to hosts that tend to interact tightly with and adhere to their symbionts (figure 3*b*), similar to the symbiotic associations between methanogens in hydrogenosomes in the cytosol of anaerobic ciliates [119]. This can, in principle, lead to a situation like that sketched in figure 3, with a prokaryotic (bacterial) symbiont residing within a prokaryotic (archaeal) host. This was a fairly radical proposal of the theory, because it did not invoke phagocytosis as the mechanism of endosymbiont entry, an aspect that drew fierce criticism from Cavalier-Smith [57]. In the meantime, examples of prokaryotes that have come to reside as stable endosymbionts within other prokaryotes have been well studied [120,121]. In those examples, the host prokaryotes are definitely not phagocytotic, so phagocytosis is clearly not a prerequisite for the establishment of intracellular symbiosis. Without question, phagocytosis greatly increases the frequency with which endosymbionts become established within eukaryotic cells [122], but—notably—none of those countless cases of phagocytosis-dependent bacterial symbiosis have ever led to anything resembling a second origin of mitochondria. Conversely, a bacterial–archaeal symbiotic association that clearly resembles a second origin of eukaryotes—from the standpoint of physiology, metabolism and the direction of gene transfer—has been described; it gave rise to the haloarchaea [123,124].

The H_2_-dependent nature of the host leads to a curious situation in phase depicted in figure 3*c*. In order to generate H_2_ for the host, the symbiont requires reduced organic compounds (fermentable organic substrates), but the host is a strict autotroph and cannot supply them in excess of its own needs because H_2_-dependent autotrophs live from gases and do not import reduced organic compounds. This phase of the symbiosis is thus unstable because the symbiont will eventually consume the host's cytosol. In order for the symbiosis to persist, either the host needs to invent importers for organics, or the symbiont's preexisting genes for importers are transferred to the host's chromosomes and can be expressed there, and the bacterial importers need to be functional in the archaeal membrane, which is true in haloarchaea [123]. Gene transfer could merely involve occasional lysis of an endosymbiont, just as it occurs in endosymbiotic gene transfer (gene transfer from organelles to the nucleus) in eukaryotes today [117], except that at this stage of the symbiosis, the host is still an archaeon and lacks a nucleus, although the bipartite cell has a bacterial endosymbiont and gene transfer from symbiont to host has commenced (figure 3*d*).

Expression of carbon importers in the host's membrane does not completely solve the problem though, because the hydrogen hypothesis posits that the host was an autotroph, hence its carbon metabolism was specialized to anabolic pathways. A good example of such enzymatic specialization is the bifunctional fructose 1,6 bisphosphate aldolase/bisphosphatase that is characteristic of archaeal autotrophs [125] but is altogether missing in eukaryotes, but many other examples of archaeal-specific enzymes of sugar-phosphate (and unphosphorylated sugar) metabolism are known [126,127]. Thus, either the enzymes of the host's anabolic metabolism need to acquire, one point mutation at a time, the substitutions required to make carbon metabolism run backwards, or, more likely and more rapidly achieved, genes for the symbiont's heterotrophic carbon metabolism are also expressed in the host's chromosomes. As in the case of the importers, this also involves straight endosymbiotic gene transfer, without targeting of the protein product to the donor symbiont, just expression in the archaeal cytosol.

This transfer does a variety of important things. First, it allows carbon to be directed to the symbiont, so that it can produce H_2_ via fermentation to satisfy the host. Second, it confers heterotrophy upon the host compartment (the cytosol), but only if transfer of the symbiont's entire glycolytic pathway is successful (the enzymatic steps all the way to pyruvate), because the first net gain of ATP in glycolysis is at the pyruvate kinase step. Third, if that occurs, it directly accounts for the bacterial origin of eukaryotic glycolytic enzymes (except enolase: [128]). No other formulation of endosymbiotic theory accounts for the observation that eukaryotes, though their ribosomes stem from archaea, have a bacterial glycolytic pathway; indeed, for other versions of endosymbiotic theory it is not even an explanandum.

Fourth, and quite unexpectedly, the selective pressure associating the two partners from the beginning and selecting the transfer of importers and glycolysis to the host compartment was the host's dependence upon H_2_ to run its carbon and energy metabolism. But the expression of genes for heterotrophic carbon flux in the host compartment supply it with reduced carbon species and ATP and there is no longer any selective pressure to maintain the host's autotrophic lifestyle, which will necessarily have involved membrane bioenergetics because all autotrophs are dependent upon chemiosmotic coupling. As a result, the host can relinquish its autotrophy; it has become a heterotroph with chimaeric chromosomes harbouring archaeal and bacterial genes, and archaeal ribosomes and glycolysis in the cytosol. In addition, the cytosol harbours a facultatively anaerobic bacterial endosymbiont with a respiratory chain and H_2_-producing fermentations (figure 3*d*) that can donate a full genome's worth of bacterial genes over and over again, replacing many indigenous archaeal pathways with bacterial counterparts, and thus transforming the archaeon from within. Part of this transformation involves the establishment of bacterial lipid synthesis (indicated in blue in figure 3); although the archaeal pathway of lipid synthesis (the mevalonate pathway) has been retained in eukaryotes [129], it is not just used for isoprene ether lipid synthesis, rather it is used for isoprenes in general, such as cholesterol (which requires only trace, that is, non-molar amounts of oxygen [130]), or for the hydrophobic tails of quinone or for dolichol phosphate.

Gene transfer from symbiont to host carries some fateful hitchhikers—self-splicing group II introns. These are indicated in figure 3 as hand-shaped structures in the symbiont's genome. Group II introns are important because their transition into spliceosomal introns is thought to have precipitated the origin of the nucleus [35]. How so? Group II introns occur in prokaryotic genomes [131,132], they are mobile, they can spread to many copies per genomes [133] and they remove themselves via a self-splicing mechanism that involves the intron-encoded maturase [134]. Their splicing mechanism is similar to that in spliceosomal intron removal [135], for which reason they have long been viewed as the precursors of both (i) spliceosomal introns and (ii) their cognate snRNAs in the spliceosome: one ‘master’ intron in the genome could provide all necessary splicing functions *in* *trans*; resident group II introns could degenerate so as to become dependent on the *trans* functions and thus to end up as small elements having conserved residues only at the splice sites and the lariat site A.

The crux of the splicing hypothesis for nuclear origins [35] is this: introns entered the eukaryotic lineage via gene transfer from the mitochondrial endosymbiont to an archaeal host (figure 3*d*), where they subsequently spread to many sites in the host's chromosomes (figure 3*e*). Evidence for this is the observation that about half of introns in eukaryotic genes are ancient, being present at positions that are conserved across divergent eukaryotic lineages, indicating their presence in the eukaryote common ancestor [35]. Once they begin to undergo the transition to spliceosomal introns a curious situation arises: splicing is slow, of the order of minutes per intron [136], while translation is fast, of the order of 10 peptide bonds per second. As the transition to spliceosomal introns set in, the host's cytosol was still a prokaryotic compartment in that there was cotranscriptional translation, with active ribosomes synthesizing proteins on nascent transcripts (figure 3*f*). That is not a problem for group II introns, which use their maturase from one ribosome passage to block the mRNA 5′ end until the intron is removed. But with the origin of fully fledged spliceosomes (symbolized as purple dumbbells in figure 3*g*) transitioning to spliceosomal splicing, nascent transcripts are translated before they can be spliced. This means that introns are translated, leading to defective gene expression at hundreds of loci simultaneously, a surely lethal condition for the host unless immediately remedied. There are a finite number of solutions to this problem, in addition to precipitating the origin of nonsense-mediated decay (nmd), a eukaryote-specific machinery that recognizes and inactivates intron-containing mRNAs [137].

One solution would be to simply remove all the introns in the chromosomes. That did not happen, because many intron positions are ancient [138,139]. Another solution would be to invent a spliceosome that is much faster than ribosomes, but that is almost like asking for a miracle, because the modern spliceosome has had more than a billion years to refine its function, but it has not become faster. Another solution would be to physically, hence spatiotemporally, separate the slow process of splicing from the fast process of translation so that the former could go to completion before the latter set in. Separation in cells usually involves membranes, and that is the central tenet of the splicing hypothesis: the initial pressure that led to selection for the nuclear membrane was to exclude active ribosomes from active chromatin (figure 3*h*), allowing the slow process of splicing to go to completion around the chromosomes, and thereby initially allowing distal diffusion, later specific export of processed mRNAs to the cytosol for translation [35]. The nuclear pore complex mediates the translocation of proteins and mRNA between the cytosol and the nucleus. Comparative genomics of nuclear pore complex proteins and proteins that make up the nucleolus shows that many of them share domains with both archaeal and bacterial proteins [140,141].

In that view, the origin of the nucleus marks the origin of a genuinely new cell compartment—not the nucleus itself, but the eukaryotic cytosol—that is free of active chromatin, where protein–protein interactions, rather than protein–DNA interactions, move to the fore in signalling and regulation, and where proteins can spontaneously aggregate and interact in such a way as to generate new structures and functions, including the true cytoskeleton and membrane traffic processes that distinguish eukaryotes from prokaryotes. A curious property of this model for the origin of the nucleus is that it only requires eukaryotes to possess a nuclear membrane when they are expressing genes, which directly points to another very curious (and vastly underappreciated) character that separates eukaryotes from prokaryotes: prokaryotes express their genes continuously during cell division, while eukaryotes shut down the expression all of their genes before chromosome partitioning and cell division. To us, this suggests an evolutionary link between splicing the splicing-dependent origin of the nucleus, the origin of genome-wide gene silencing mechanisms [142], which generally involve chemical modifications of chromatin and histones, and the origin of the eukaryotic cell cycle.

This set of events leads to a bipartite cell (figure 3*h*) (i) that requires a nucleus in order to express genes, (ii) that has retained archaeal ribosomes in the cytosol as a vestige of the host, (iii) that has bacterial energy metabolism both in the cytosol and in the mitochondrion, (iv) that has lost all electron-transfer phosphorylation functions in the plasma membrane, (v) that has nonetheless retained the archaeal ATPase, which however now operates backwards to acidify the vacuole, and (vi) that has typical eukaryotic features. It is true that many theories for eukaryote origin surveyed here address many of the same aspects, but what everyone has overlooked for the now nearly 50 years since Margulis revived endosymbiotic theory [88] is that the myriad inventions that distinguish eukaryotes from prokaryotes do not come for free. The origin of eukaryotic novelties had an energetic price, and that price was paid by mitochondria [34]. The internalization of bioenergetic membranes in eukaryotes frees them from the bioenergetic constraints that keep prokaryotes prokaryotic in organization. Since the late 1990s, there has been a growing realization that all eukaryotes have or had mitochondria, but it had not been clear why that is the case, until the calculations were done [34]. That puts the mitochondrial symbiosis at the very beginning of eukaryogenesis.

## 6. Rounding out the picture: the plastid

**Figure 4.**
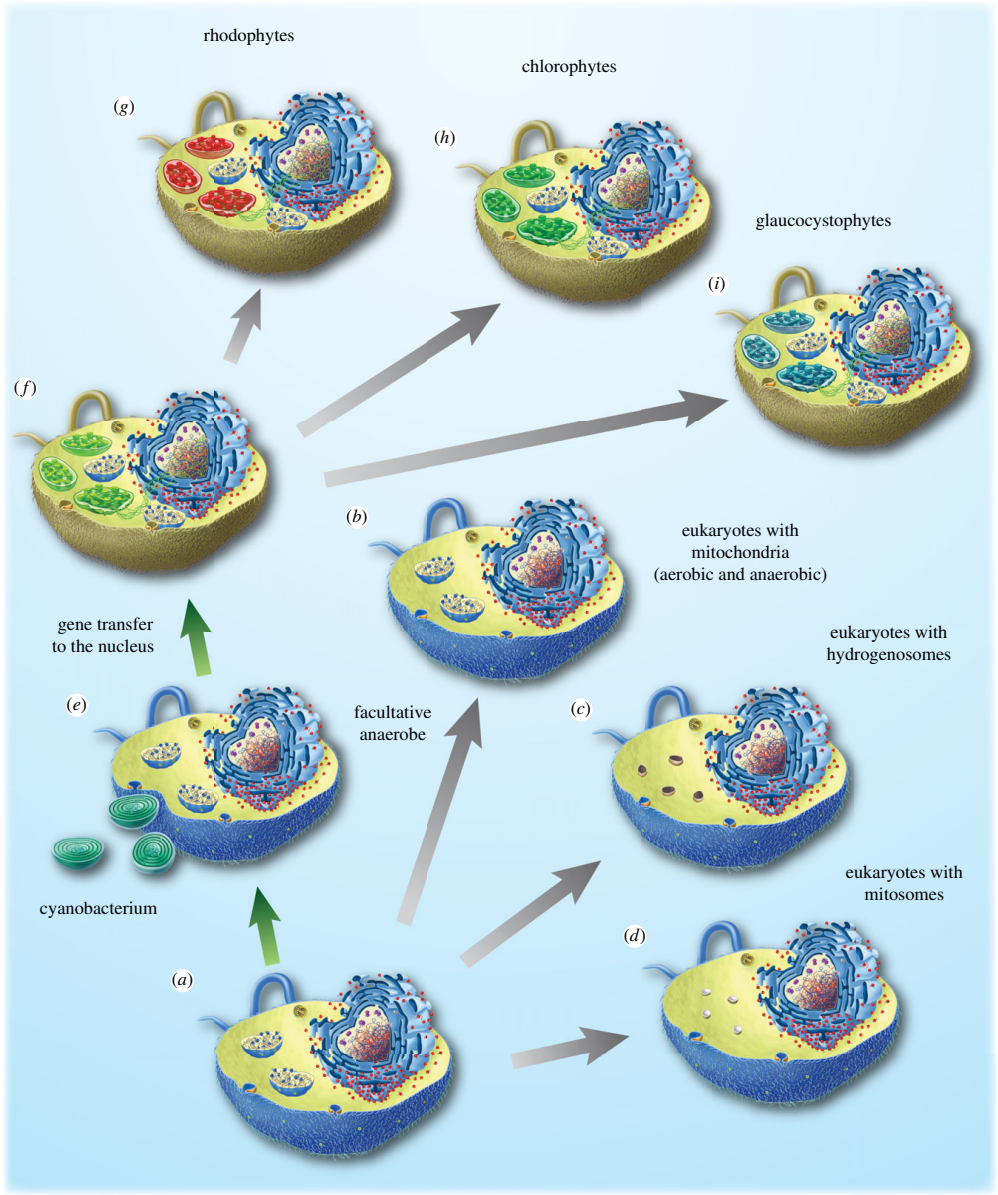
Evolution of anaerobes and the plastid. (*a*–*d*) Diversification of the mitochondria-containing ancestor to eukaryotes containing specialized forms of the organelle, hydrogenosomes, mitosomes and anaerobic mitochondria. See also [21,143]. (*e*,*f*) Primary symbiotic origin of a plastid involving a cyanobacterium in a facultative anaerobic host (see text), followed by gene transfer to the nucleus resulting in a plastid-bearing ancestor. See also [144]. (*g*–*i*) Diversification of the plastid-bearing ancestor to glaucocystophytes, chlorophytes and rhodophytes. See also [25].

Of course, there was one additional and crucial prokaryotic endosymbiont in eukaryotic history: a cyanobacterium that became the plastid. This is outlined in figure 4. The ancestral eukaryote was, seen from the standpoint of energy metabolism [21], a facultative anaerobe. It underwent specialization to aerobic and anaerobic environments in multiple independent lineages, giving rise to eukaryotes specialized to either aerobic or anaerobic environments [143], as well as giving rise to facultative anaerobes, like *Euglena* [21,145,146] or *Chlamydomonas* [147–149]. The prevalence of enzymes for anaerobic energy metabolism in eukaryotes in general [143], and in particular among algae like *Chlamydomonas* [149], together with the circumstance that they use the same enzymes that *Trichomonas* and *Giardia* use to survive under anaerobic conditions, not to mention their conservation in *Cyanophora* [150], lead to a novel inference of some interest: the host for the origin of plastids was a facultative anaerobe.

The origin of plastids has been the subject of several recent papers [41,81,82,151]. In terms of endosymbiotic theory, the situation is clear: a eukaryote that already possessed a mitochondrion—a facultative anaerobe, as we just pointed out—obtained a cyanobacterium as an endosymbiont (figure 4*e*); possible metabolic contexts [152] for that symbiosis could have involved carbohydrate produced by the plastid, oxygen produced by the plastid [25], nitrogen supplied by the plastid [153] or a combination thereof. Although the phylogenetic affinity of the cyanobacterium that became the plastid is complicated by the circumstance that prokaryotes avidly undergo LGT, current analyses point to large-genomed, nitrogen-fixing forms [151,154]. Similar to the case for mitochondria, many genes were transferred from the endosymbiont to the host's chromosomes [144], which in the case of plastids were surrounded by a nucleus (figure 4*f*). The origin of protein import machineries of organelles played an important role, both in the case of mitochondria [155] and in the case of plastids [156], because it allowed the genetic integration of host and endosymbiont while allowing the endosymbiont to maintain its biochemical identity. The three lineages of algae harbouring primary plastids—the chlorophytes, the rhodophytes and the glaucocystophytes—diverged early in plastid evolution (figure 4*g–i*). At least two secondary endosymbioses involving green algae occurred [157–159], and at least one, but possibly more, secondary symbioses involving red algal endosymbionts occurred during evolution, whereby protein import probably also played an important role in the establishment of red secondary endosymbioses [82].

Since the inception of endosymbiotic theory by Mereschkowsky [13,15], the founding event that gave rise to primary plastids has been seen as the incorporation of the cyanobacterial endosymbiont. Over the past few years, a variant of endosymbiotic theory has, however, emerged that sees the plastid symbiosis as beginning with a chlamydial infection of a eukaryotic cell, an infection that was cured by the cyanobacterium. The chlamydial story for plastid origin developed slowly but has made its way into prominent journals lately [160]. There are several very severe problems with the chlamydia story, as several authors have recently pointed out [41,82,152,161,162]. Perhaps the most serious problem is that the gene trees upon which the current versions of the chlamydial theory are based do not say what the proponents of the chlamydial theory claim. This is shown in new analyses both by Deschamps [162], who provides an excellent historical overview of the chlamydial theory, and by Domman *et al*. [152]. Both papers show that the suspected chlamydia connection to plastid origin is founded in phylogenetic artefacts—trees that do not withstand critical methodological inspection. Because of phylogenetic factors and because of LGT among prokaryotes, trees can be misleading in the context of inferring endosymbiont origins [41], and it is prudent to look at other kinds of evidence as well. As it concerns the origin of mitochondria, Degli-Esposti [163] surveyed the components of proteobacterial membrane bioenergetics and inferred that the ancestor of mitochondria was methylotrophic.

## 7. Organelles have retained genomes (why?)

An important component of endosymbiotic theory is the circumstance that organelles have retained genomes. The observation that organelles had DNA at all was one of the key observations that supported endosymbiotic theory in the first place [102]. Indeed, several autogenous (non-endosymbiotic) alternatives to the endosymbiont hypothesis were designed specifically to explain the existence of DNA in organelles [94–96]. With very few important exceptions (that prove the rule, explained below), organelles have retained DNA.

Why have organelles retained DNA? The answer to that question is satisfactorily explained by only one theory: John F. Allen's CoRR hypothesis (co-location for redox regulation) [164,165]. It posits that organelles have retained genomes so that individual organelles can have a say in the expression of components of the respiratory and photosynthetic electron transport chains in order to maintain redox balance in the bioenergetic membrane. The CoRR hypothesis directly accounts for the observation that plastids and mitochondria have converged in gene content to encode almost exclusively genes involved in their respective electron transport chains, and components of the ribosome necessary to express them in the organelle. It has also recently come to the attention of some of us interested in endosymbiosis that plastids and mitochondria (and to some extent nucleomorphs) have furthermore converged in gene content to encode the same set of ribosomal proteins [38]. A compelling explanation for the otherwise puzzling and long overlooked convergence for ribosomal protein content in plastid and mitochondrial genomes is ribosome assembly; the process of ribosome biogenesis requires that some proteins need to be coexpressed in the same compartment as their nascent rRNAs [38]. The convergence observed in gene content in plastid and mitochondrial genomes is striking.

One of the burgeoning strengths of Allen's CoRR hypothesis for the evolutionary persistence of organelle genomes concerns its predictions with regard to hydrogenosomes. Hydrogenosomes have more or less everything that mitochondria have, but they have lost the respiratory chain in their inner membrane. CoRR posits the selective pressure to maintain organelle DNA to be the necessity to maintain redox balance. Some readers might ask: What is redox balance? Redox balance refers to the smooth flow of electrons through the electron transport chain. The concept of redox balance applies both to mitochondria and to chloroplasts, because both have electron transport chains that generate proton gradients to drive their respective ATPase. In both electron transport chains, quinols and quinones are an essential component. These membrane soluble electron carriers can transfer electrons non-enzymatically to O_2_, generating the superoxide radical (O_2_^−^), which is the starting point for reactive oxygen species (ROS) [166]. If the flow of electrons through the bioenergetic membrane (the inner mitochondrial membrane or the thylakoid) is impaired, for example, because downstream components in the chain are present in insufficient amounts, or because upstream components in the chain are too active, then the steady-state quinol concentration increases (quinols are the reduced form of the quinones) and the quinols generate ROS. If an organelle relinquishes its electron transport chain, then there is, according to CoRR, no need to retain the genome, it can become lost, and precisely this has happened in hydrogenosomes, in no less than four independent lineages: trichomonads, ciliates, fungi and amoeboflagellates [21]. Other theories for organelle genome persistence, for example the theory that organelles encode hydrophobic proteins [167], do not make that prediction.

## 8. Eukaryotes tug and twist the archaeal tree

There is currently much buzz about the possibility that a group of crenarchaeotes, the TACK superphylum (for Thaumarchaeota, Aigarchaeota, Crenarchaeota and Korarchaeota) might harbour the closest ancestors of the host that acquired the mitochondrion. Several different trees that address the issue have appeared recently ([30,31,50,51]; discussed in [168]). One aspect of those trees that has so far gone unmentioned is that trees that place the eukaryotic informational genes within the crenarchaeotes also root the archaea either with euryarchaeotes basal [50], within the euryarchaeotes [169] or within the methanogens [31,50–52]. Also, archaeal trees that do not include eukaryotes also tend to root the archaea within methanogens or within euryarchaeotes [30,51,52,170]. There are a number of traits that make methanogens excellent candidates for the most ancient among the archaeal lineages [171], methanogenesis is currently the oldest biological process for which there is evidence in the geological isotope record, going back some 3.5 Ga [172], and microbiologists considered methanogenesis to be one of the most primitive forms of prokaryotic metabolism even before archaea were discovered [173]. A methanogenic ancestry of archaea makes sense in many ways.

In line with that, abiotic (geochemical) methane production occurs spontaneously at serpentinizing hydrothermal vents [174–176] (for a discussion of serpentinization, see [177]). Of all naturally occurring geochemical reactions currently known, only the process of serpentinization at hydrothermal vents involves exergonic redox reactions that emulate the core bioenergetic reactions of some modern microbial cells [177–181]. The point is this: if the ancestral state of archaeal carbon and energy metabolism is methanogenesis, then all archaea are ancestrally methanogenic and ancestrally hydrogen dependent. This is relevant for models of eukaryote origins that involve anaerobic synthrophy (a hydrogen-dependent archaea host for the origin of mitochondria), because then hydrogen dependence becomes a very widespread trait affecting the evolution of all archaeal lineages, including those that gave rise to the eukaryotic host lineage.

Indeed, recent findings have it that many archaeal lineages stem from methanogenic ancestors via gene transfers [124]. In particular, the origin of haloarchaea is noteworthy because it entailed exactly the same physiological transformation (from strictly anaerobic H_2_-dependent chemolithoautotroph to facultatively anaerobic heterotroph) as the hydrogen hypothesis posits for the origin of eukaryotes [123], and the mechanism underlying that transformation—gene transfer from bacterium to archaeon—is the same as in the hydrogen hypothesis. The main difference between the origin of the respiratory chain of haloarchaea and of mitochondria is that the former operates in an archaeal cytoplasmic membrane whereas the latter operates in the internalized bioenergetic membranes of mitochondria within eukaryotic cells [123]. It is precisely that difference, however, that separates the eukaryotes from the prokaryotes in terms of the metabolic energy available to drive the evolution of novel protein families and thus novel cell biological traits [34].

Thus, as the position of eukaryotes starts to come into focus within the archaeal tree, so does the position of the root among archaea, and multiple evolutionary transitions from an ancestrally H_2_-dependent state seems to be a recurring theme within the archaea, with gene transfers from bacteria providing the physiological capabilities to access electron and energy sources other than H_2_. Early archaeal evolution and the origin of eukaryotes are ancient events, so ancient that they push phylogenetic methods to their limits, and possibly beyond. The book of early evolution holds many exciting chapters, and the origin of eukaryotes is clearly one of the most crucial, because eukaryotes—and only eukaryotes, the cells that have mitochondria—brought forth genuinely complex life.
